# Tetrac and NDAT Induce Anti-proliferation via Integrin αvβ3 in Colorectal Cancers With Different *K-RAS* Status

**DOI:** 10.3389/fendo.2019.00130

**Published:** 2019-03-12

**Authors:** Yu-Tang Chin, Zong-Rong He, Chi-Long Chen, Hsiao-Ching Chu, Yih Ho, Po-Yu Su, Yu-Chen S. H. Yang, Kuan Wang, Ya-Jung Shih, Yi-Ru Chen, Jens Z. Pedersen, Sandra Incerpi, André Wendindondé Nana, Heng-Yuan Tang, Hung-Yun Lin, Shaker A. Mousa, Paul J. Davis, Jacqueline Whang-Peng

**Affiliations:** ^1^Taipei Cancer Center, Taipei Medical University, Taipei, Taiwan; ^2^Cancer Center, Wan Fang Hospital, Taipei Medical University, Taipei, Taiwan; ^3^Department of Pediatrics, E-Da Hospital, Kaohsiung, Taiwan; ^4^School of Medicine, I-Shou University, Kaohsiung, Taiwan; ^5^School of Medicine, Taipei Medical University, Taipei, Taiwan; ^6^Division of Medical Imaging, E-Da Cancer Hospital, I-Shou University, Kaohsiung, Taiwan; ^7^School of Pharmacy, Taipei Medical University, Taipei, Taiwan; ^8^Graduate Institute of Nanomedicine and Medical Engineering, College of Biomedical Engineering, Taipei Medical University, Taipei, Taiwan; ^9^Joint Biobank, Office of Human Research, Taipei Medical University, Taipei, Taiwan; ^10^Department of Biology, University of Rome Tor Vergata, Rome, Italy; ^11^Department of Sciences, Roma Tre University, Rome, Italy; ^12^Graduate Institute of Cancer Biology and Drug Discovery, College of Medical Science and Technology, Taipei Medical University, Taipei, Taiwan; ^13^Pharmaceutical Research Institute, Albany College of Pharmacy and Health Sciences, Rensselaer, NY, United States; ^14^TMU Research Center of Cancer Translational Medicine, Taipei Medical University, Taipei, Taiwan; ^15^Traditional Herbal Medicine Research Center of Taipei Medical University Hospital, Taipei Medical University, Taipei, Taiwan; ^16^Department of Medicine, Albany Medical College, Albany, NY, United States

**Keywords:** perfusion bellows cell culture system, colorectal cancer cells, anticancer, phosphoERK1/2, NDAT, tetrac, integrin αvβ3

## Abstract

Colorectal cancer is a serious medical problem in Taiwan. New, effective therapeutic approaches are needed. The selection of promising anticancer drugs and the transition from pre-clinical investigations to clinical trials are often challenging. The deaminated thyroid hormone analog (tetraiodothyroacetic acid, tetrac) and its nanoparticulate analog (NDAT) have been shown to have anti-proliferative activity *in vitro* and in xenograft model of different neoplasms, including colorectal cancers. However, mechanisms involved in tetrac- and NDAT-induced anti-proliferation in colorectal cancers are incompletely understood. We have investigated possible mechanisms of tetrac and NDAT action in colorectal cancer cells, using a perfusion bellows cell culture system that allows efficient, large-scale screening for mechanisms of drug actions on tumor cells. Although integrin αvβ3 in *K-RAS* wild type colorectal cancer HT-29 cells was far less than that in *K-RAS* mutant HCT116 cells, HT-29 was more sensitive to both tetrac and NDAT. Results also indicate that both tetrac and NDAT bind to tumor cell surface integrin αvβ3, and the agents may have different mechanisms of anti-proliferation in colorectal cancer cells. *K-RAS* status appears to play an important role in drug resistance that may be encountered in treatment with this drug combination.

## Introduction

The well-demonstrated receptor of thyroid hormone on the extracellular domain of plasma membrane integrin αvβ3 is mainly expressed on tumor cells and dividing blood vessel cells. Steroid hormones (1–3) and thyroid hormones (L-thyroxine, T_4_, and 3,5,3′-triiodo-L-thyronine, T_3_) are able to bind to integrin αvβ3 on cells (4–11). The interaction between thyroid hormone and integrin αvβ3 (11) has been demonstrated to promote cancer cell proliferation in various types of cancer cells, including breast cancer (12, 13), lung cancer (10, 14), glioma cells (15, 16), myeloma cells (17), pancreatic cancer (18), and colorectal cancer cells (6, 7, 13, 19). Crosstalk between epidermal growth factor (EGFR) and the integrin has been reported to be involved in regulation of cancer cell proliferation (7, 13). Signal transduction mechanisms mediate the promotion of cancer cell proliferation by thyroid hormone and such mechanisms can be blocked by tetraiodothyroacetic acid (tetrac) (7, 13), a deaminated analog of thyroid hormone.

Tetrac has been shown to compete with thyroid hormone, e.g., L-thyroxine (T_4_) for the iodothyronine receptor on integrin αvβ3. Tetrac inhibits binding of thyroid hormones, thus inhibiting the downstream signal transduction pathways of nongenomically initiated effects of thyroid hormone (4, 7, 19–21). Tetrac also has actions at the receptor that are independent of the functions of thyroid hormone, for example, modulation of angiogenesis by multiple mechanisms and regulation of tumor cell metabolism (22). Its interaction with integrin αvβ3 permits tetrac to modify differentially regulated gene expression that is related to cancer cell survival pathways. Tetrac up-regulates expression of pro-apoptotic *BcL-x* short form (12), anti-angiogenic thrombospondin 1 (*THBS1*) and other pro-apoptotic genes (4). In addition, tetrac downregulates transcription of several families of anti-apoptotic genes. A nano-formulation of tetrac, nano-diamino-tetrac (NDAT), has been shown to act primarily at the cell surface and does not enter the nucleus when it does enter the cell.

Recently we have studied the anti-proliferative effects of tetrac, NDAT, and their combinations with other anticancer drugs on colorectal cancer cells (4, 7, 13, 19–21). In combination with resveratrol, NDAT downregulated resveratrol-induced ribonucleotide reductase regulatory subunit M2 (*RRM2*) gene expression *in vivo* and potentiated the anticancer effect of the stilbene (20). In addition, tetrac enhanced nuclear abundance of chibby family member 1 (CBY1), a nuclear β-catenin antagonist, which is a protein that may compromise nuclear β-catenin-dependent gene expression and proliferation (6). Gefitinib-induced anti-proliferation in gefitinib-resistant colorectal cancer cells is restored by NDAT; the mechanism involves inhibition of beta-galactoside alpha-2, 6-sialyltransferase 1 (ST6Gal1) activity and PI3K activation (7). These observations indicate that added or enhanced effects are obtained with combinations of tetrac or NDAT and other chemotherapeutic agents.

In the current report, we investigated mechanisms by which tetrac- and NDAT induced anti-proliferation in colorectal cancer cells. In addition, we report studies conducted to define the different gene profiles induced by tetrac and NDAT in colorectal cancer cell lines. Finally, using a novel perfusion bellows cell culture system, we have distinguished the mechanisms by which tetrac or NDAT work on human colorectal cancer cells with different *K-RAS* status.

## Materials and Methods

### Cell Cultures

Human colorectal cancer cell lines HT-29 (ATCC® HTB-38™) and HCT 116 (ATCC® CCL-247™) were obtained from American Type Culture Collection (ATCC) (Manassas, VA, USA). Cells were maintained in RPMI-1640 medium (Life Technologies Corp. Carlsbad, CA, USA) supplemented with 10% FBS and grown under 5% CO_2_/95% air at 37°C routinely. Prior to treatments, cells were washed with phosphate buffered saline (PBS) and then serum-free medium was added for starvation for 48 h. Then, the serum-free medium was replaced by 5% stripped FBS containing medium at the initiations of studies.

### Pharmacodynamics

Anti-proliferative effects of tetrac and NDAT were defined in a well-established perfusion bellows cell culture system (13, 23). At the outset, 5 × 10^7^ cells were seeded in perfusion bellows cell culture system and incubated at 37°C overnight. Then polymer flakes were harvested, trypsinized, and cells were collected and counted. The number of original cells attached to flakes was 0.5 × 10^7^ cells/bottle. Cell cultures were refreshed with 1% stripped FBS-containing medium. Tetrac or NDAT was added in a medium bottle to the final concentrations indicated in the Results section. Specific concentration of tetrac and NDAT were chosen according to the physiological concentration of T_4_ (10^−7^ M) as described previously (24–26). The samples of cell-bearing flakes were then treated as indicated, and cells were harvested at timeframe indicated, trypsinized, and collected for counting. The cell cultures were refreshed with 10% hormone-stripped FBS containing medium.

### Quantitative Real-Time PCR (QPCR)

Total RNA was extracted and genomic DNA was eliminated with the Illustra RNAspin Mini RNA Isolation Kit (GE Healthcare Life Sciences, Buckinghamshire, UK). One microgram of DNase I-treated total RNA was reverse-transcribed with a RevertAid H Minus First Strand cDNA Synthesis Kit (Life Technologies Corp.) into cDNA and used as the template for real-time PCR reactions and analysis. The real-time PCR reactions were performed using QuantiNovaTM SYBR® Green PCR Kit (QIAGEN, Valencia, CA, USA) on a CFX Connect™ Real-Time PCR Detection System (Bio-Rad Laboratories, Inc., Hercules, CA, USA). This involved an initial denaturation at 95°C for 5 min, followed by 40 cycles of denaturing at 95°C for 5 s and combined annealing/extension at 60°C for 10 s, as described in the manufacturer's instructions. The primer sequences were: *Homo sapiens* integrin, alpha v (*ITG* α*v*), forward 5′-TCCGATTCCAAACTGGGAGC-3′ and reverse 5′-AAGGCCACTGAAGATGGAGC-3′ (Accession No.: NM_002210.4); *Homo sapiens* integrin, beta 3 (*ITG* β*3*), forward 5′-CTGGTGTTTACCACTGATGCCAAG-3′ and reverse 5′-TGTTGAGGCAGGTGGCATTGAAGG-3′ (Accession No.: NM_000212.2); *Homo sapiens* caspase 2, apoptosis-related cysteine peptidase (*CASP2*), forward 5′-GCATGTACTCCC ACCGTTGA-3′ and reverse 5′-GACAGGCGGAGCTTCTTGTA-3′ (Accession No.: NM_032982.3); *Homo sapiens* v-myc avian myelocytomatosis viral oncogene homolog, (*c-Myc*), forward 5′-TTCGGGTAGTGGAAAACCAG-3′ and reverse 5′-CAGCAGCTCGAATTTCTTCC-3′ (Accession No.: NM_002467); *Homo sapiens* p53-inducible gene 3 (*PIG3*) / tumor protein p53 inducible protein 3 (*TP53I3*), forward 5′-TTGAGGCATCTGGACATGTG-3′ and reverse 5′-GGGTCAATCCCTCTGGGATAG-3′ (Accession No.: NM_004881.4); *Homo sapiens* tumor protein p53 (*p53*), forward 5′-AAGTCTAGAGCCACCGTCCA-3′ and reverse 5′-CAGTCTGGCTGCCAATCCA-3′ (Accession No.: NM_000546.5); *Homo sapiens* cyclin-dependent kinase inhibitor 1A (*p21*), forward 5′- CTGGGGATGTCCGTCAGAAC-3′ and reverse 5′-CATTAGCGCATCACAGTCGC-3′ (Accession No.: BT006719.1); *Homo sapiens* BCL2-associated agonist of cell death (*BAD*), forward 5′- CTTTAAGAAGGGACTTCCTCGCC-3′ and reverse 5′-AAGTTCCGATCCCACCAGGA-3′ (Accession No.: NM_032989.2); *Homo sapiens* vascular endothelial growth factor A (*VEGF-A*), forward 5′-TACCTCCACCATGCCAAGTG-3′ and reverse 5′-GATGATTCTGCCCTCCTCCTT-3′ (Accession No.: NM_001204384.1); *Homo sapiens* programmed death ligand 1 (*PD-L1*) (*CD274*), forward 5′-GTTGAAGGACCAGCTCTCCC-3′ and reverse 5′-ACCCCTGCA TCCTGCAATTT-3′ (Accession No. NM_014143.3); *Homo sapiens* thrombospondin 1, (*THBS1*), forward 5′-ATCCTGGACTCGCTGTAGGT-3′ and reverse 5′-GTCATCGTCCCTTTCGGTGT-3′ (accession no.: BC136470.1); and *Homo sapiens* 18S ribosomal RNA (*18S*), forward 5′-GTAACCCGTTGAACCCCATT-3′ and reverse 5′-CCATCCAATCGGTAGTAGCG-3′ (accession no. NR_003286). The relative gene expression normalized to the internal control 18S rRNA was calculated based on the ΔΔCT method and the fidelity of the PCR reactions was determined with melting temperature analysis.

### Western Blotting

Western blotting analyses were conducted followed routine protocol described previously (6, 7, 19–21, 23). Consistent amount of protein samples were resolved on a 10% sodium dodecyl sulfate polyacrylamide gel (SDS-PAGE). A 20 μg quantity of protein was loaded in each well with 5x sample buffer, and the protein samples were resolved with electrophoresis at 100 V for 2 h. The resolved proteins were transferred from the polyacrylamide gel to Millipore Immobilon-PSQ Transfer PVDF membranes (Millipore, Billerica, MA, USA) with the Mini Trans-Blot® Cell (Bio-Rad Laboratories). The membranes were blocked with a solution of 2% FBS in Tris-buffered saline and incubated with primary antibodies to pERK1/2 (GeneTex, Inc., Hsinchu, Taiwan), Cyclin D1 (Santa Cruz Biotechnology, Inc., Dallas, TX, USA), ERK1/2 (Santa Cruz Biotechnology), and α-Tubulin (Novus Biologicals, Littleton, CO, USA) at 4°C overnight and washed; the proteins were detected with HRP-conjugated secondary antibodies and Immobilon™ Western HRP Substrate Luminol Reagent (Millipore). Images of the western blots were visualized and recorded with an Amersham Imager 600 (GE Healthcare, Chicago, IL, USA). The blots were quantified using ImageJ software (National Institutes of Health, Bethesda, MD, USA).

### Flow Cytometry Analysis

HCT116 cells and HT-29 cells were harvested from polymer flakes by trypsinization, washed with PBS, and resuspended in 1 mL PBS (1 × 10^6^ − 5 × 10^6^ cells). To quantify the expression of integrin αvβ3 on the cell membrane, cells were fixed with 70% ethanol for 30 min at 4°C. Cells were washed in PBS three times and then resuspended in 100 μL PBS with 1% bovine serum albumin (BSA) at a concentration of 5 × 10^6^ cells per mL. Cells were incubated with mouse monoclonal anti-integrin αvβ3 antibody (1:50; Santa Cruze Biotechnology Inc.) at room temperature for 1 h. Cells were further incubated with an Alexa-647-labeled goat anti-rabbit antibody (1:200, GeneTex International Corporation, Hsinchu City, Taiwan) at room temperature for 30 min in the dark. Flow cytometry was carried out on a FACSCalibur™ (Becton Dickinson) instrument, using CellQuest software to determine the expression of integrin αvβ3. Fluorescence-activated cell sorting (FACS) analysis used integrin αvβ3-Alexa-647. Relative percentages of integrin αvβ3-positive cells were calculated from FL-3 histograms using ModFit LT software.

### Data Analysis and Statistics

Data are presented as the mean ± S.E. The data were analyzed using IBM SPSS Statistics software version 19.0 (SPSS, Inc., Chicago, IL, USA). One-way analysis of variance (ANOVA) with Duncan's *post-hoc* test was used to analyze the differences between experimental groups followed by a paired Student's *t*-test. Student's *t*-tests for paired data were also used in some cases as indicated. *p* < 0.05 was considered statistically significant.

## Results

### Different Expression Levels of Integrin αvβ3 Are Present on the Cell Surface of Colorectal Cancer Cells

**Figure 1:**
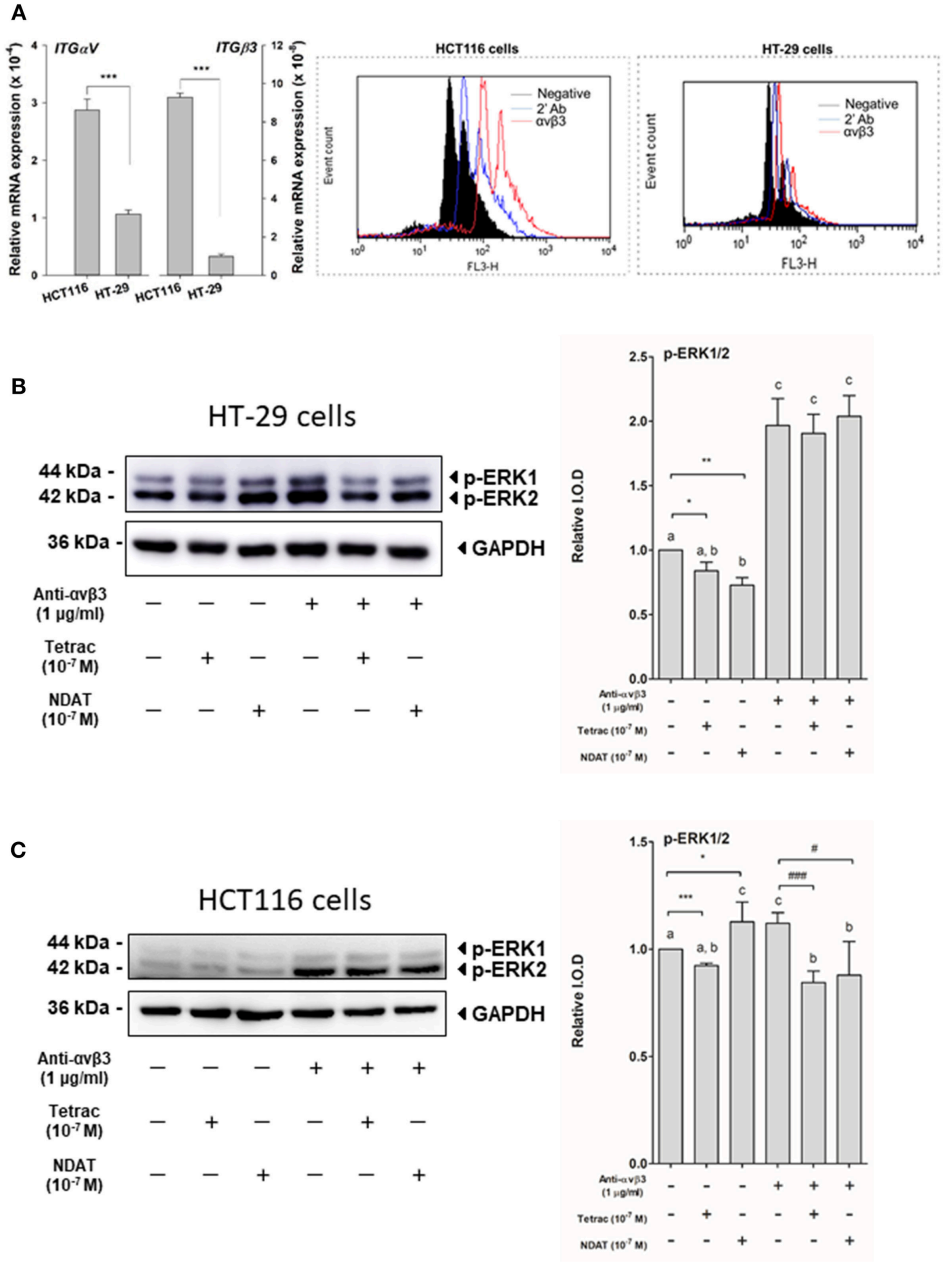
Cell surface integrin αvβ3 is the binding site of tetrac and NDAT in colorectal cancer cells. **(A)** Eighty-five percent confluent colorectal cancer cells, HT-29 cells, and HCT116 cells, grown in 10-cm Petri dishes were harvested for studies of QPCR and flow cytometry analysis of integrin αvβ3. For studies of ERK1/2 activation, HT-29 cells and HCT116 cells seeded in 10-cm Petri dishes were pretreated with 1 μg/mL of anti-integrin αvβ3 antibody for 30 min and then treated with either 10^−7^ M tetrac or 10^−7^ M NDAT for 30 min. Total proteins were extracted, then Western blot analyses were conducted. **(B)** Activation of ERK1/2 was induced by NDAT but not tetrac in HT-29 cells. **(C)**. Activation of ERK1/2 was inhibited by tetrac and NDAT in HCT116 cells. Pretreatment of anti-integrin αvβ3 antibody reversed their effects. Number of independent experiments. *N* = 3. (Data are expressed as mean ± SD; ^*^p < 0.05, ^**^p < 0.01, ^***^p < 0.001, compared with untreated control; ^#^*p* < 0.05, ^###^*p* < 0.001, compared with anti-integrin αvβ3 antibody treatment. a-c: the subsets after *post hoc* analysis after the significant differences were obtained using one-way ANOVA).

To evaluate the expression of integrin αvβ3 in the colorectal cancer cell lines HCT116 and HT-29, studies of QPCR and flow cytometry analysis of integrin αvβ3 were conducted. The expressions of *ITG*α*V* and *ITG*β*3* in HCT116 cells were significantly higher than that in HT-29 cells (2.7 fold in *ITG*α*V* and 9.4 fold in *ITG*β*3*) (Figure 1A, left panel). The expression of integrin αvβ3 on the cell surface of HCT116 cells (35.6%) was also notably greater than that on HT-29 cells (12.6%) (Figure 1A, right panel).

### Both Tetrac and NDAT Bind to Cell Surface Integrin αvβ3 in Colorectal Cancer Cells

To determine whether the signal transduction pathways activated by tetrac and NDAT are integrin αvβ3-dependent, colorectal cancer *K-RAS*-mutant HCT116 cells and *K-RAS*-wild type HT-29 cells were set in 10 mL Petri dishes and starved with serum-free media for 2 days. Prior to starting each experiment, cells were re-fed with fresh medium. Cells were treated with 1 μg/mL anti-integrin αvβ3 30 min before treatment of either 10^−7^ M tetrac or NDAT for 30 min. Total proteins were extracted and Western blot analyses were conducted for pERK1/2 study. Tetrac inhibited activation of ERK1/2, but NDAT increased activated ERK1/2 in HT-29 cells (Figure 1B). On the other hand, tetrac and NDAT inhibited constitutively activated ERK1/2 in HCT116 cells (Figure 1C). However, this effect was partially removed by anti-integrin αvβ3 antibody pretreatment (Figures 1B,C).

**Figure 2:**
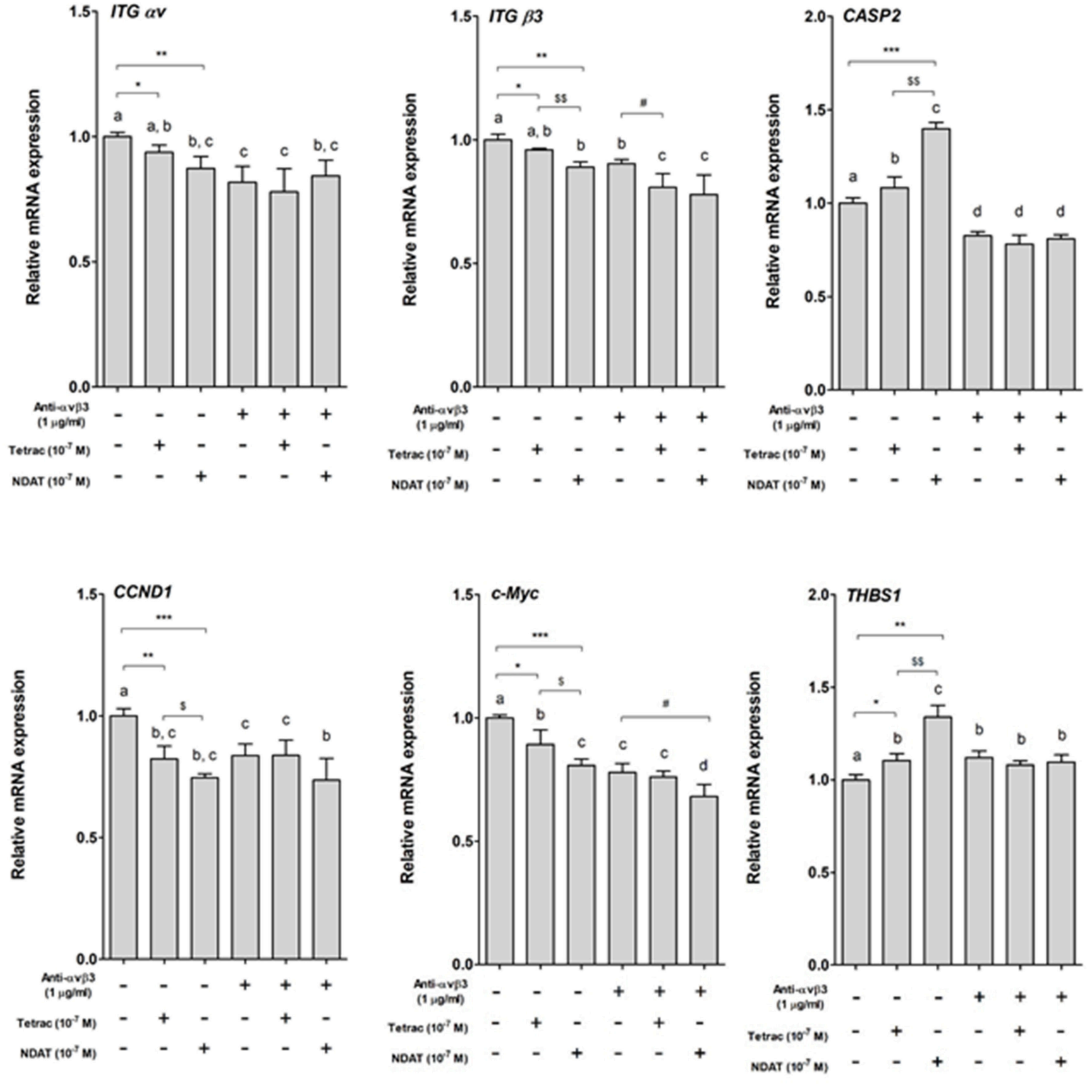
Tetrac and NDAT via cell surface integrin αvβ3 regulate gene expression in colorectal cancer cells. HCT116 cells seeded in 6-well plates were pretreated with 1 μg/mL of anti-integrin αvβ3 antibody for 30 min and then treated with either 10^−7^ M tetrac or 10^−7^ M NDAT for 24 h. Total RNA was extracted and QPCR was conducted. *N* = 3. (Data are expressed as mean ± SD; ^*^p < 0.05, ^**^p < 0.01, ^***^p < 0.001, compared with untreated control; ^*$*^*p* < 0.05, ^*$$*^*p* < 0.01, compared with tetrac group; #*p* < 0.05, compared with anti-integrin αvβ3 antibody treatment. a–d: the subsets after *post hoc* analysis after the significant differences were obtained using one-way ANOVA).

Parallel studies were conducted to define the expression of specific genes controlled by tetrac or NDAT. Colorectal cancer HCT116 cells were set in 6-well trays and starved with serum-free media for 2 days. Prior to starting each experiment, cells were re-fed with fresh medium. Cells were treated with 1 μg/mL anti-integrin αvβ3 for 30 min prior to treatment with 10^−7^ M tetrac or NDAT for 24 h. Total RNA was extracted and qPCR was conducted for integrin αv (*ITG* α*v*), integrin β3 (*ITG* β*3*), *CASP2, CCND1, c-Myc*, and *THBS1*. Results shown in Figure 2 indicate that both tetrac and NDAT inhibited expression of *CCND1* and *c-Myc*, but promoted expression of *CASP2* and *THBS1*. The effect of NDAT was much greater than that of tetrac. However, pretreatment of cells with anti-integrin αvβ3 blocked the effect of tetrac and NDAT (Figure 2). These results suggest that both tetrac and NDAT bind to integrin αvβ3 to induce downstream signals that play an important role in tetrac/NDAT-dependent cellular activities.

### Tetrac and NDAT Induce Anti-proliferation in Colorectal Cancer Cells With Different *K-RAS* Status

**Figure 3:**
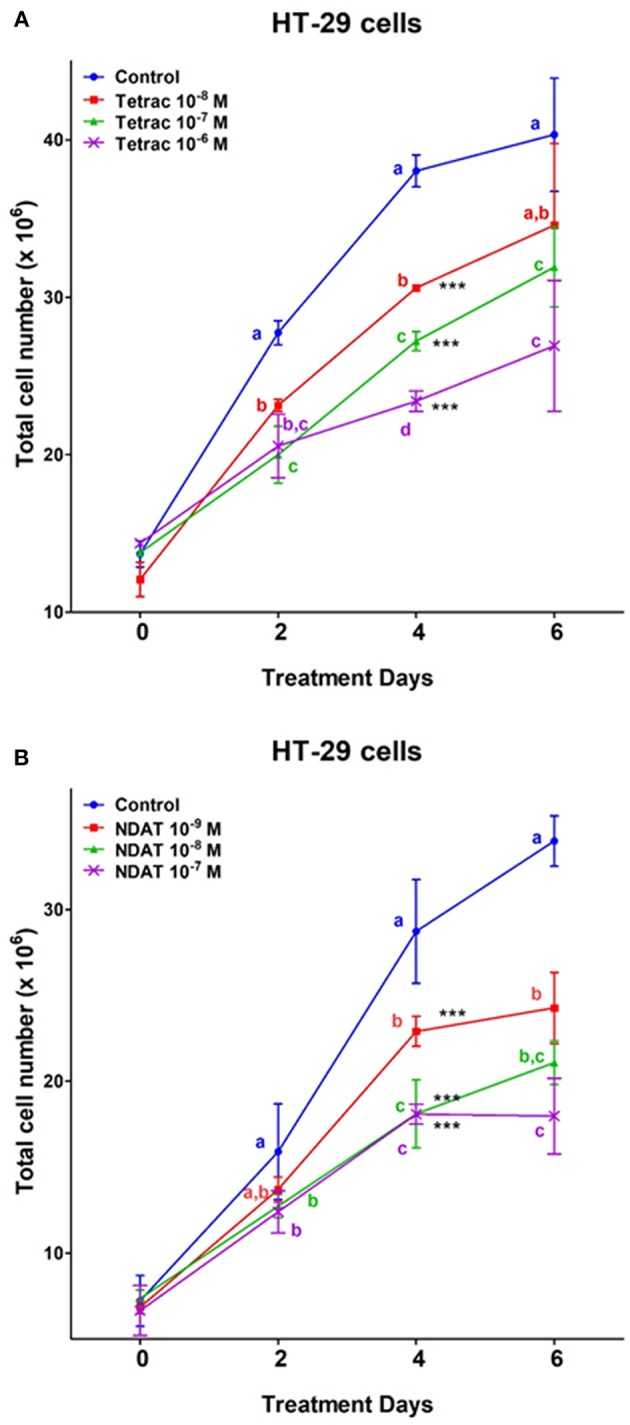
Tetrac **(A)** and NDAT **(B)** induce anti-proliferation of *K-RAS* wild-type colorectal cancer HT-29 cells. HT-29 colorectal cancer cells were grown in the perfusion bellows system and treated with tetrac (10^−8^ to 10^−6^ M) or NDAT (10^−9^ to 10^−7^ M) for 6 days. After incubated in the treatment, cells were isolated and subjected to analysis by cell count, *N* = 3. Data are expressed as mean ± SD; ^***^p < 0.001, compared with untreated control. a–c: the subsets after *post hoc* analysis after the significant differences were obtained using one-way ANOVA.

Two colorectal cancer cell lines were seeded in the perfusion bellows cell culture system. Different concentrations of tetrac (10^−8^, 10^−7^, and 10^−6^ M) or NDAT (10^−9^, 10^−8^, and 10^−7^ M) were added to medium bottles. Tetrac of various concentrations (10^−8^, 10^−7^, and 10^−6^ M) significantly reduced cell number of *K-RAS*-wild type HT-29 cells after 4 days of treatment, as did NDAT (10^−8^ and 10^−7^ M). NDAT (10^−8^ M) reduced 37% of cell number after 4 days of treatment, but tetrac (10^−7^ M) only reduced 28.4% of cell number compared to untreated control (Figure 3).

**Figure 4:**
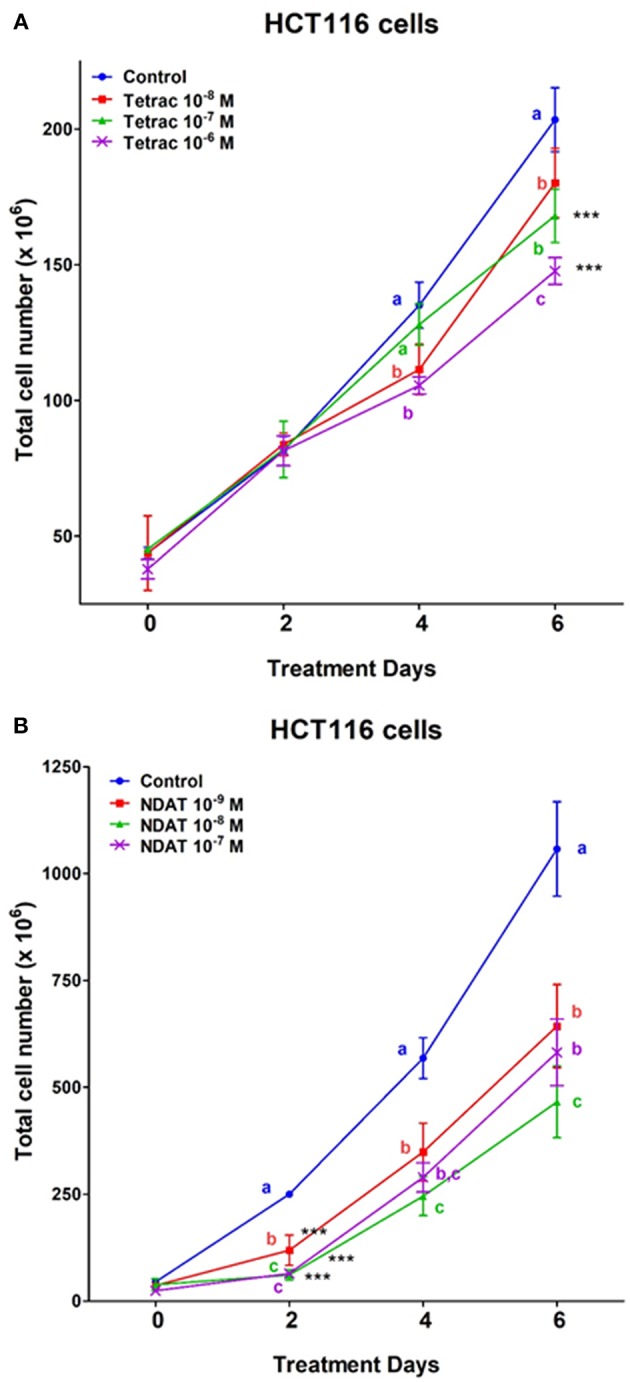
Tetrac **(A)** and NDAT **(B)** induce anti-proliferation of *K-RAS*-mutant colorectal cancer HCT116 cells. HCT116 colorectal cancer cells were grown in the perfusion bellows system and treated with tetrac (10^−8^ to 10^−6^ M) or NDAT (10^−9^ to 10^−7^ M) for 6 days. After incubated in the treatment, cells were isolated and subjected to analysis by cell count. *N* = 3. Data are expressed as mean ± SD; ^***^p < 0.001, compared with untreated control. a–c: the subsets after *post hoc* analysis after the significant differences were obtained using one-way ANOVA.

After 6 days of treatment, tetrac (10^−8^ and 10^−7^ M) significantly reduced cell number of *K-RAS*-mutant HCT116 cells (Figure 4). On the other hand, different concentrations of NDAT (10^−9^, 10^−8^, and 10^−7^ M) inhibited cancer cell proliferation significantly after 2 days of treatment. NDAT (10^−9^ M) reduced 52.5% of cell number at day 2, but tetrac (10^−8^ M) reduced 11.5% of cell number at day 5 compared to untreated control (Figure 4).

**Figure 5:**
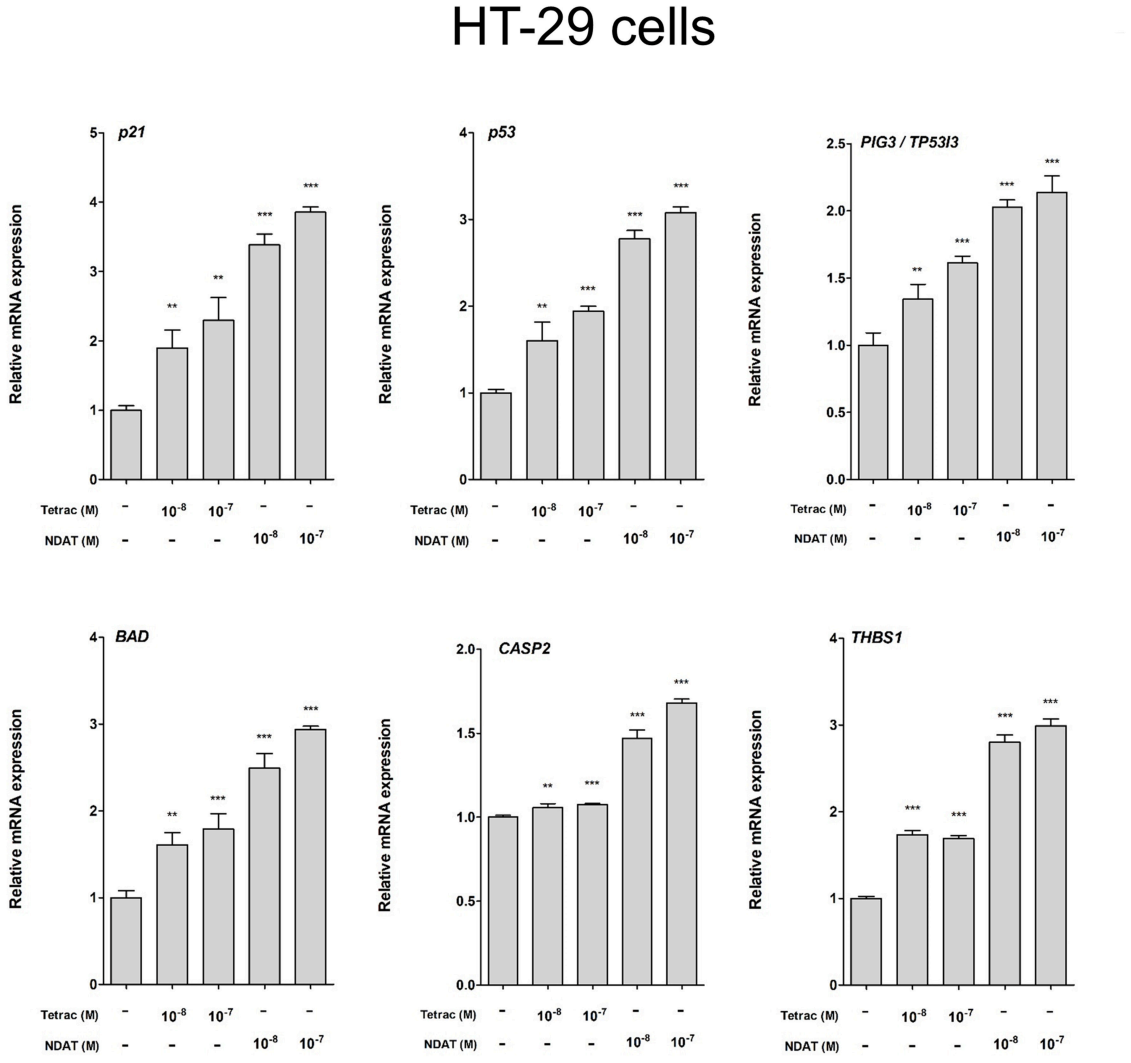
Tetrac and NDAT suppress expression of proliferative and metastasis-related genes in HT-29 cancer cells. HT-29 cells were seeded in 6-well plates and treated with different concentrations of 10^−8^ and 10^−7^ M tetrac or NDAT for 24 h. Cells were harvested and total RNA was extracted. qPCR experiments were conducted to examine expression of anti-proliferative genes (*p21, p53*, and *PIG3*), apoptotic genes (*BAD* and *CASP2*) and metastasis-related genes (*THBS1*). *N* = 6. (Data are expressed as mean. ± SD; ^*^p < 0.05, ^**^p < 0.01, ^***^p < 0.001, compared with untreated control).

Cells treated with tetrac and NDAT were also prepared for flow cytometry studies. Results shown in Figure 5 indicate that tetrac induced cancer cell arrested in G2/M stage. However, low concentration 10^−8^ M NDAT induced G2/M arrest and 10^−7^ M NDAT shut down DNA synthesis.

### NDAT and Tetrac Modulate Expression of Different Genes in Colorectal Cancer Cells

**Figure 6:**
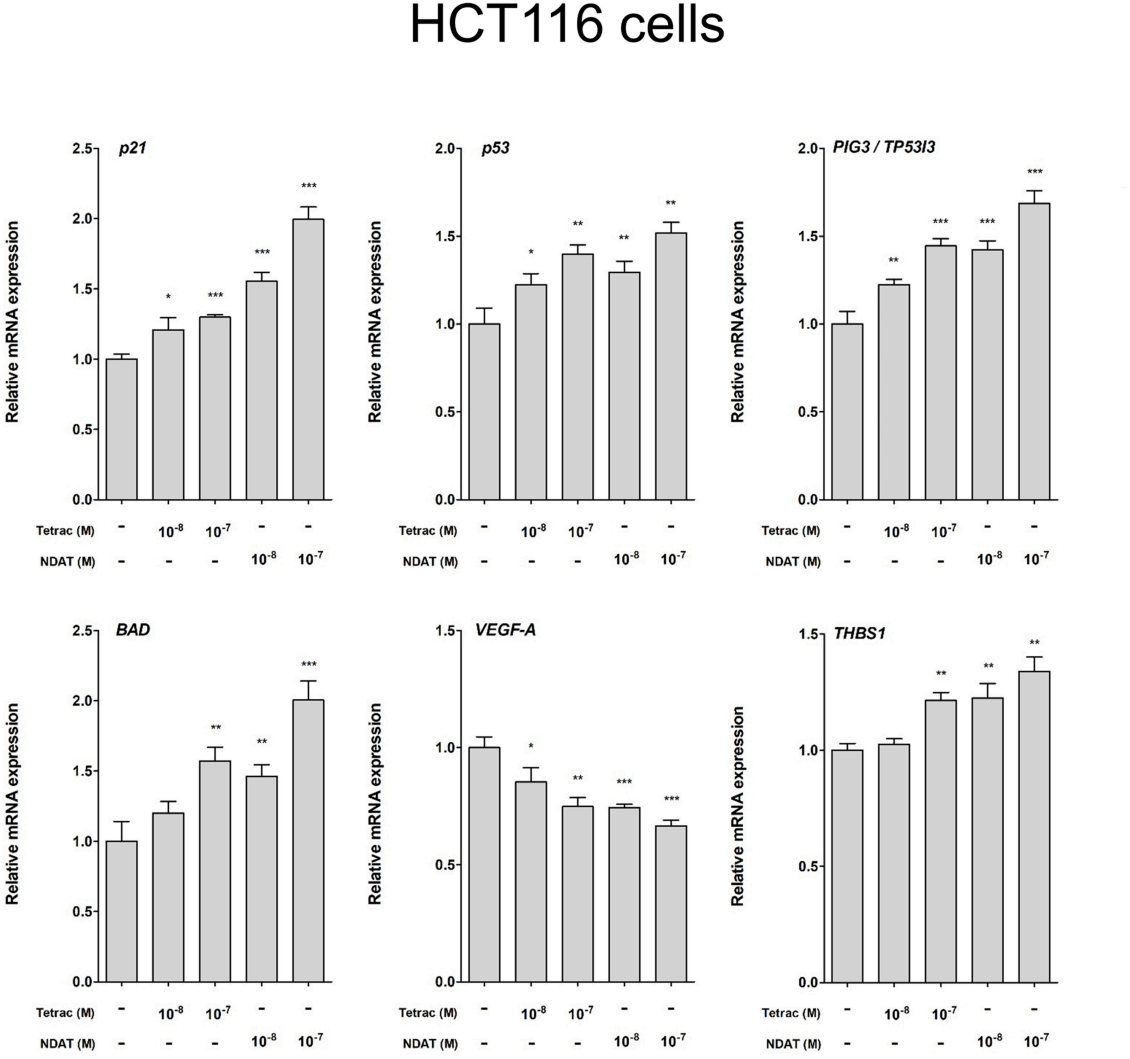
Tetrac and NDAT suppress expression of proliferative and metastasis-related genes in HCT116 cancer cells. HCT116 cells were seeded in 6-well plates and treated with different concentrations of 10^−8^ and 10^−7^ M tetrac or NDAT for 24 h. Cells were harvested and total RNA was extracted. Studies of qPCR were conducted to examine expression of anti-proliferative genes (*p21, p53*, and *PIG3*), apoptotic gene (*BAD*), and metastasis-related genes (*VEGF-A* and *THBS1*). *N* = 6. (Data are expressed as mean ± SD; ^*^p < 0.05, ^**^p < 0.01, ^***^p < 0.001, compared with untreated control).

We observed previously that treatment with tetrac or NDAT results in different gene expression profiles in MDA-MB-231 cells and in medullary thyroid carcinoma cells (24). In order to define the gene transcription profiles induced by tetrac and by NDAT in colorectal cancer cells with different *K-RAS* status, HT-29 cells and HCT116 cells were treated with 10^−8^ and 10^−7^ M tetrac or NDAT, respectively for 24 h. RNA was extracted from the harvested cells at the end of treatment for qPCR studies. Treatment with NDAT (10^−8^ M) for 24 h increased expression of *p53* and p53-responsible genes such as *p21, PIG3 (TP53I3), BAD*, and *CASP2* in HT-29 cells significantly (Figure 5). In addition, NDAT increased expression of *THBS1*. Protein TSP1 is an endogenous suppressor of angiogenesis and is invariably suppressed in cancer cells. Although tetrac also increased expression of those genes, the effect was 10-fold less (Figure 5). In contrast, tetrac (10^−8^ and 10^−7^ M) significantly increased expression of *p21, p53, PIG3, BAD*, and *THBS1* genes in HCT116 cells (Figure 6). However, 10^−8^ M tetrac did not significantly inhibit expression of *VEGF-A*, which plays an important role in HCT116 cells metastasis (Figure 6). On the other hand, NDAT also increased expression of the mentioned genes and significantly decreased expression of *VEGF* at 10^−8^ M.

## Discussion

The perfusion bellows cell culture studies we describe here provide useful pharmacodynamic information of the application of new drugs or combinations of various agents *in vitro* to human cancer cell lines (13). Pharmacodynamic modeling based on the expected pharmacokinetics of a drug permits—in the perfusion bellows cell culture system– the understanding of dose-response relationship of antineoplastic agents over a very wide range of concentration in *vitro* and can support translation from *in vitro* models to animal models and ultimately human clinical trials.

Via binding to the thyroid hormone receptor on plasma membrane integrin αvβ3 (27, 28), NDAT induces signal transduction and biological activities similar to those of unmodified tetrac (Figures 1, 2), but with desirable effects on cell survival pathway genes that differ from the parent thyroid hormone analog (Figures 2, 5, 6) (5, 24). Studies indicated that NDAT but not tetrac activated ERK1/2 in *K-RAS*-wild type colorectal cancer HT-29 cells (Figure 1A), which confirms previous results that NDAT activates ERK1/2 in HT-29 cells (24). On the other hand, both tetrac and NDAT inhibited ERK1/2 activation in *K-RAS*-mutant HCT-116 cells (Figure 1B). Interestingly, NDAT is more sensitive to the inhibitory effect of pretreatment of cells with of anti-integrin αvβ3 (Figure 2). Both tetrac and NDAT induced anti-proliferation in colorectal cancer *K-RAS*-wild type HT-29 cells and in *K-RAS*-mutant HCT116 cells (Figures 3, 4). The growth-inhibitory effects of tetrac and NDAT on HCT116 colon cancer cell xenografts are evident in 2–4 d after the onset of treatment (6, 7, 20). The anti-proliferative effect of tetrac and NDAT on cancer cells in the perfusion bellows cell culture system was seen at 3 d after the start of treatment (Figures 3, 4). Pharmacodynamics of anti-proliferative activities *in vitro* of tetrac and NDAT in colorectal cancer cells were compared in the perfusion bellows cell culture system. Results revealed that NDAT had a higher potency than tetrac as an anti-proliferative agent (Figure 3). We have previously shown that the anticancer effects of tetrac and NDAT in colorectal cancer HCT116 cell xenografts are well-established within 3 d after onset of drug administration (6, 7). Although inhibitory effects of tetrac and NDAT in the perfusion bellows cell culture system are limited to suppression of cell proliferation, these results in the perfusion system reproduce findings obtained earlier in the same cells in xenografts. Tetrac-/NDAT-induced anti-tumor effects in xenografts have been shown to involve both primary effects on tumor cell proliferation and anti-angiogenesis effect (6, 7). Results in these sets of studies similarly demonstrate that the anticancer effects of tetrac/NDAT occur first as anticancer cell proliferation.

The inhibition of HT-29 cell growth by NDAT and tetrac was comparable at a higher drug concentration (10^−7^ M), but there was increased sensitivity to NDAT at a lower drug concentration of the used agents (Figure 3). In addition to the anti-proliferative effects initiated at the cell surface integrin receptor, tetrac may penetrate into cells to exert low-grade thyromimetic (proliferative) effects in the nucleus of treated colorectal cancer cells. Therefore, the net anti-proliferative effect of tetrac decreases. On the other hand, NDAT does not gain access to the cell nucleus and shows a more robust anti-proliferative effect.

In addition to their anti-proliferative effects on cancer cells, tetrac and NDAT have been shown to enhance anti-cancer growth by other anticancer drugs. Cetuximab (Erbitux®) inhibits *K-RAS* wild-type but not *K-RAS* mutant colorectal cancer cell growth (19). The combination of tetrac and cetuximab significantly reduced cell proliferation compared to cetuximab alone in *K-RAS* mutant HCT 116 cells, but not in *K-RAS* wild type COLO 205 cells (19). NDAT also rescued the anti-proliferative effect of cetuximab in both colorectal cancer cell lines (19). These results suggest that tetrac and NDAT may be used in the clinic in the future to treat *K-RAS*-mutant colorectal cancer patient. However, present studies indicated that NDAT was much more effective than tetrac (Figures 5, 6). Furthermore, NDAT reversed *K-RAS* mutant-dependent cetuximab resistance. In addition, xenograft weights in NDAT-treated alone animals are not significantly decreased compared to those in untreated control [result not shown (6, 7)]. Therefore, NDAT alone or combination with low dosage of cetuximab may be a new chemotherapeutic approach in the future.

Studies have shown that tetrac affects only XIAP gene expression (12) in breast cancer cells. On the other hand, NDAT downregulates expression of apoptosis inhibitors *XIAP*, myeloid cell leukemia sequence 1 (*MCL1*), and apoptosis-promoting genes such as *CASP2* and *BCL2L14* (12). Expression of the angiogenesis inhibitor thrombospondin 1 (*THBS1*) gene is increased by both unmodified tetrac and NDAT, as is the expression of *CBY1*, a nuclear inhibitor of catenin activity (12). The majority of differentially regulated *K-RAS*-oncogene family members and the epidermal growth factor receptor gene are downregulated by NDAT, but not by tetrac (12). β-Catenin belongs to a class of catenins that have been shown to play roles in cell-cell adhesion. It also has transcription functions. Various cancers such as those of the colorectum, breast, and ovary have been shown to contain mutant and overexpressed β*-catenin* genes. CBY1, an inhibitor of nuclear functions of β-catenin, is upregulated by NDAT. Like β-catenins, integrin αvβ3 participates in cellular adhesion complexes. NDAT downregulates expression of the gene for integrin β3 monomer. Since integrin αvβ3 has been shown to involve metastasis and migration, this may be a desirable action of NDAT at αvβ3 in cancer cells.

NDAT also downregulates expression of the genes for α*-catenins, CTNNA1* and *CTNNA2* (8). Mutation of CTNNA2 is associated with tumor invasiveness and thus inhibition of transcription of the gene is desirable. The nonmutated gene product of CTNNA1 can function as a tumor invasion suppressor, but mutation is associated with gastrointestinal tract and other cancers.

Thyroid hormone induces expression of MMP-2 and MMP-9 in myeloma cells and colorectal cancer cells (7). This action is inhibited by tetrac and NDAT; this implies that αvβ3 can be involved in the contribution of matrix metalloproteinases to cancer-relevant angiogenesis and to tumor invasiveness. Tetrac and NDAT were able to inhibit constitutive expression of *VEGF-A* (Figure 6). Tetrac and NDAT also increased transcription of thrombospondin 1 (*THBS1, TSP1*), which suppresses angiogenesis.

In summary, tetrac and NDAT via integrin αvβ3 regulate thyroid hormone-dependence of various independent cancer cell activities. NDAT is much more potent than tetrac and has a broader range of transcriptional actions in cancer cells that may be clinically desirable.

## Author Contributions

Y-TC, Z-RH, C-LC, KW, H-YL, and JW-P: conceptualizing research idea. H-CC, YH, P-YS, Y-CY, and KW: developing methodologies. P-YS, Y-CY, KW, Y-JS, Y-RC, AWN, and H-YT: performing experiments. JZP, SI, Y-JS, Y-RC, AWN, and H-YT: collecting and analyzing data. JZP and SI: Interpreting data. H-YL and PJD: writing manuscript. SAM, PJD, and JZP: proofreading and editing.

### Conflict of Interest Statement

Co-authors SAM and PJD are co-inventors of NDAT and stockholders in a company that is commercializing the agent. The remaining authors declare that the research was conducted in the absence of any commercial or financial relationships that could be construed as a potential conflict of interest.
