# Corrigendum: Tetrac and NDAT Induce Anti-proliferation via Integrin αvβ3 in Colorectal Cancers With Different *K-RAS* Status

**DOI:** 10.3389/fendo.2019.00241

**Published:** 2019-04-09

**Authors:** Yu-Tang Chin, Zong-Rong He, Chi-Long Chen, Hsiao-Ching Chu, Yih Ho, Po-Yu Su, Yu-Chen S. H. Yang, Kuan Wang, Ya-Jung Shih, Yi-Ru Chen, Jens Z. Pedersen, Sandra Incerpi, André Wendindondé Nana, Heng-Yuan Tang, Hung-Yun Lin, Shaker A. Mousa, Paul J. Davis, Jacqueline Whang-Peng

**Affiliations:** ^1^Taipei Cancer Center, Taipei Medical University, Taipei, Taiwan; ^2^Cancer Center, Wan Fang Hospital, Taipei Medical University, Taipei, Taiwan; ^3^Department of Pediatrics, E-Da Hospital, Kaohsiung, Taiwan; ^4^School of Medicine, I-Shou University, Kaohsiung, Taiwan; ^5^School of Medicine, Taipei Medical University, Taipei, Taiwan; ^6^Division of Medical Imaging, E-Da Cancer Hospital, I-Shou University, Kaohsiung, Taiwan; ^7^School of Pharmacy, Taipei Medical University, Taipei, Taiwan; ^8^Graduate Institute of Nanomedicine and Medical Engineering, College of Biomedical Engineering, Taipei Medical University, Taipei, Taiwan; ^9^Joint Biobank, Office of Human Research, Taipei Medical University, Taipei, Taiwan; ^10^Department of Biology, University of Rome Tor Vergata, Rome, Italy; ^11^Department of Sciences, Roma Tre University, Rome, Italy; ^12^Graduate Institute of Cancer Biology and Drug Discovery, College of Medical Science and Technology, Taipei Medical University, Taipei, Taiwan; ^13^Pharmaceutical Research Institute, Albany College of Pharmacy and Health Sciences, Rensselaer, NY, United States; ^14^TMU Research Center of Cancer Translational Medicine, Taipei Medical University, Taipei, Taiwan; ^15^Traditional Herbal Medicine Research Center of Taipei Medical University Hospital, Taipei Medical University, Taipei, Taiwan; ^16^Department of Medicine, Albany Medical College, Albany, NY, United States

**Keywords:** perfusion bellows cell culture system, colorectal cancer cells, anticancer, phosphoERK1/2, NDAT, tetrac, integrin αvβ3

In the original article, we neglected to include the funder “E-Da Hospital, EDAHP107018” to Z-RH.

The authors apologize for this error and state that this does not change the scientific conclusions of the article in any way. The original article has been updated.

